# Unstable carotid artery plaque: new insights and controversies in diagnostics and treatment

**DOI:** 10.3325/cmj.2016.57.311

**Published:** 2016-08

**Authors:** Karolina Skagen, Mona Skjelland, Mahtab Zamani, David Russell

**Affiliations:** 1 Department of Neurology, University Hospital Rikshospitalet, Oslo, Norway; 2 Institute of Clinical Medicine, University of Oslo, Oslo, Norway; * These authors contributed equally

## Abstract

Cardiovascular disease is estimated to be the leading cause of death, globally causing 14 million deaths each year. Stroke remains a massive public health problem and there is an increasing need for better strategies for the prevention and treatment of this disease. At least 20% of ischemic strokes are thromboembolic in nature, caused by a thromboembolism from an atherosclerotic plaque at the carotid bifurcation or the internal carotid artery. Current clinical guidelines for both primary and secondary prevention of stroke in patients with carotid stenosis caused by atherosclerotic plaques remain reliant on general patient characteristics (traditional risk factors for stroke) and static measures of the degree of artery stenosis. Patients with similar traditional risk factors, however, have been found to have different risk of stroke, and it has in recent years become increasingly clear that the degree of artery stenosis alone is not the best estimation of stroke risk. There is a need for new methods for the assessment of stroke risk to improve risk prediction for the individual patient. This review aims to give an overview of new methods available for the identification of carotid plaque instability and the assessment of stroke risk.

Cardiovascular disease (CVD) is estimated to be the leading cause of death globally, causing 14 million deaths each year (1). It is also the leading cause of long-term disability in older adults, and consequently a substantial contributor to health care costs. Stroke remains a massive public health problem and there is an increasing need for better strategies for the prevention and treatment of this disease. At least 20% of ischemic strokes are thromboembolic in nature (2), caused by a thromboembolism from an atherosclerotic plaque at the carotid bifurcation or the internal carotid artery. Such strokes have been shown to be preventable by surgical removal of the plaque with carotid endarterectomy (CEA) or stenting (3,4).

Current clinical guidelines for both primary and secondary prevention of stroke in patients with carotid stenosis caused by atherosclerotic plaques remain reliant on general patient characteristics (traditional risk factors; hypertension, smoking, diabetes, and hypercholesterolemia) and static measures of the degree of artery stenosis (5). Patients with similar traditional risk factors, however, have been found to have different risk of stroke (6), and it has in recent years become increasingly clear that the degree of artery stenosis alone is not the best estimation of stroke risk. There is a need for new methods for the assessment of stroke risk to improve risk prediction for the individual patient. Research has shown that the properties of the plaque itself are important for its stability and the risk of stroke, and that inflammation plays an important pathogenic role in all stages of plaque development, including rupture (7-9). There has therefore, over the last decade, been a paradigm shift in the image-based risk stratification of carotid disease from the static measurement of carotid artery stenosis to the characterization of the dynamic biological processes occurring within carotid plaques. The focus of atherosclerosis research has also broadened to include not only the study of a single atherosclerotic carotid plaque, but also the identification and assessment of generalized inflammation as well as the association between systemic inflammation and cardiovascular risk.

The most recent revision of the American College of Cardiology/American Heart Association (AHA) Task Force on Practice Guidelines continued the emphasis on the use cholesterol level-lowering drugs to reduce cardiovascular risk based on epidemiologically defined traditional risk factors (10). However, this 2013 task force acknowledged that other treatment approaches, including the use of carotid plaque burden features to determine individual risk and modify treatment therapy may be important, but are not yet evaluated in randomized clinical trials.

The limitations of current clinical guidelines for primary and secondary prevention of stroke for patients with carotid artery stenosis become particularly evident with reference to two clinical patient groups:

(i) Patients with asymptomatic carotid artery stenosis: where the identification and treatment of risk factors remains the hallmark of risk assessment and treatment. However, despite optimal medical management aimed at identifiable risk factors, patients with asymptomatic carotid stenosis develop an ischemic stroke. The Asymptomatic Carotid Stenosis and Risk of Stroke Trial (ACSRS) is a large prospective study on patients with asymptomatic carotid stenosis (ACS) undergoing medical intervention (6). ACSRS has shown that not all patients with ACS carry the same risk of stroke. Specifically the severity of carotid stenosis, a history of contralateral transient ischemic attack (TIA), and several carotid plaque features on ultrasonography could stratify patients into groups of varying annual stroke risk from less than 1% to greater than 10%. There are two ongoing large randomized clinical trials evaluating medical therapy vs surgical/endovascular intervention in patients with ACS (Carotid Revascularization Endarterectomy vs Stenting Trial [CREST-2] (11) and Asymptomatic Carotid Surgery Trial [ACST-2]) (12). Both will include an imaging sub-study to evaluate the role of vulnerable plaque imaging in asymptomatic patients with greater than 70% carotid stenosis.

(ii) Patients with symptomatic stenosis causing mild-moderate carotid artery stenosis where clinical guidelines are based on studies more than 15 years old (NASCET 1999, ESCT 1998) (3,4) in which the degree of stenosis was used to predict stroke risk and also the main criteria for selecting patients for surgical removal of plaque.

This review will focus on new methods available for the identification of carotid plaque instability and the assessment of stroke risk.

## The pathophysiology of atherosclerosis

Atherosclerotic lesions develop slowly over many years, passing through several stages. Risk factors for atherosclerosis such as hypertension, diabetes, smoking, hypercholesterolemia, genetic disposition, infection, and elevated homocysteine can activate pro-inflammatory and anti-thrombotic responses that induce endothelial dysfunction (13). Inflammation and oxidative stress mediate these risk factors, especially oxidized low-density lipoprotein (ox-LDL, due to hypercholesterolemia), which can activate the endothelium, leading to increased permeability and endothelial dysfunction (13). These dysfunctional endothelial cells then express adhesion molecules (vascular cell adhesion molecule-1, VCAM-1, and p-selectin) allowing the adherence of monocytes, T-lymphocytes, and platelets to the endothelium, which then become pro-inflammatory and pro-thrombotic (14). Monocytes are recruited into the intima (by monocyte chemo attractant protein-1, MCP-1) and differentiate into macrophages in response to macrophage colony-stimulating factors (M-CSF) and other pro-inflammatory mediators. Macrophages ingest ox-LDL by receptor-mediated phagocytosis, which promotes the transformation of macrophages into lipid-laden foam cells leading to retention of LDL particles and immune cells in the intimal layer of the vessel wall. This accumulation of lipids and associated immune cells, the “fatty streaks,” are asymptomatic and non-stenotic, and are histologically the earliest sign of atherosclerosis. Macrophages and other immune cells release cytokines, chemokines, and growth factors, which mediate further leukocyte recruitment leading to further inflammation and over time the development of an atherosclerotic plaque. Proliferation and migration of smooth muscle cells (SMCs) from the adventitia to intima lead to the development of the fibrous cap, which surrounds the atherosclerotic lesion. In addition to SMCs, the fibrous cap consists of connective tissue, such as collagen, which may be weakened by matrix metalloproteinases (MMPs). The atherosclerotic process progresses with apoptosis of the SMCs and macrophages mainly regulated by cytotoxic T-cells, leading to the formation of a lipid-rich necrotic core and increased plaque instability.

As the plaque core becomes necrotic it contains increasing amounts of cellular debris, crystalline cholesterol, and inflammatory cells, especially macrophage foam cells. The necrotic core becomes covered by the fibrous cap, which also contains inflammatory cells mainly in the “shoulder” region (the portion of the plaque lateral to the lipid core), where T-cells, mast cells, and especially macrophages have a tendency to accumulate (15,16). Advanced plaque lesions become increasingly complex with calcification, new vessel formation, thinning of the fibrous cap, and eventually plaque rupture. The strength of the fibrous cap, which is very important for plaque stability, is determined by the activity of the different pro-inflammatory and anti-inflammatory cytokines/mediators within the plaque. This determines the balance between production (transforming growth factor-β [TGF-β]) and breakdown (MMPs and interferon-γ [IFN-γ]) of collagen.

In the event of plaque rupture, the plaque content including the thrombogenic lipid core is exposed to blood containing platelets and coagulation factors with a resultant high risk of thrombus formation. Cerebrovascular symptoms are the result of these thrombi causing vessel occlusion at the plaque location and/or emboli that are carried in the blood stream to the brain where they occlude cerebral blood vessels.

## The unstable carotid plaque, inflammation, and features of plaque instability

Traditionally, atherosclerosis has been viewed as a slowly progressive, cholesterol storage disease involving the passive accumulation of cholesterol debris in the arterial wall. However, atherosclerosis is now accepted as a generalized inflammatory disease. In fact, inflammation plays an important pathogenic role in all stages of atherosclerosis including the destabilization of atherosclerotic plaques (7-9). Histological features associated with this destabilization and plaque instability are a large lipid rich necrotic core (LRNC), intra plaque hemorrhage (IPH), and thinning of the fibrous cap (FC) (17-19). Stable plaques in contrast are characteristically more calcified with a thicker fibrous cap.

## Ultrasound imaging

Carotid ultrasonography allows the measurement of intima-media thickness (IMT), detection of plaque, description of plaque morphology, and estimation of luminal stenosis, and assessment of plaque echogenicity (20,21). Plaque echogenicity, total plaque area, and plaque surface irregularities have all been suggested as ultrasound markers of unstable plaques and increased stroke risk (22-25). Total plaque area has been shown to be a more sensitive and reliable measure of atherosclerotic burden than IMT and plaque (26).

## Intima media thickness (IMT)

IMT is the measurement of the thickness of tunica intima and tunica media, the innermost two layers of the wall of an artery made by carotid ultrasound. Increased carotid IMT in patients over the age of 50 has been shown to be predictive of future cardiovascular events (27). However, the clinical usefulness of measuring the progression or potential decrease in the IMT as a surrogate endpoint to assess drug efficacy in clinical trials or in clinical management in cardiovascular disease is unclear. This is because the annual change in carotid IMT often estimated to be from 0.01 to 0.04 mm in health and disease is lower than the current-generation ultrasound pixel resolution of 0.1 to 0.2 mm, making it very difficult to follow carotid IMT change in individual patients short term (28-31).

## Plaque echogenicity

**Figure 1:**
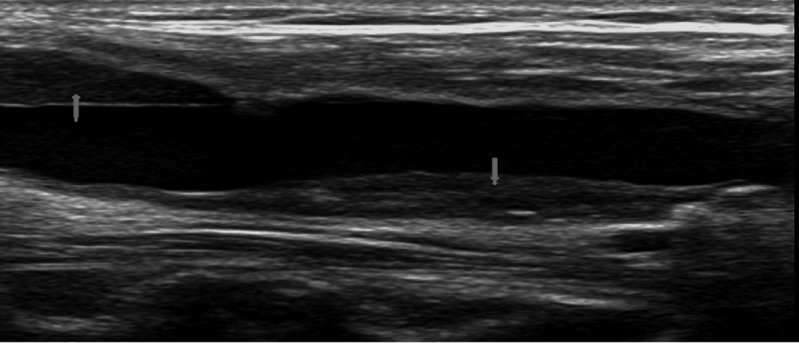
Carotid ultrasound showing echolucent plaques. Plaque in the far wall of the common carotid artery (arrow pointing down) and in the near wall at the carotid bifurcation (arrow pointing up).

The level of plaque echogenicity reflects plaque content (32,33). Although the association between echolucent plaques, plaque instability, and cerebrovascular events is not fully understood, there is a probable association between echolucency and inflammatory burden, with macrophages contributing to plaque destabilization in echolucent lesions (34,35). Echolucent carotid artery plaques (rich in lipids and/or intraplaque hemorrhage) are associated with increased risk of stroke, independent of the degree of artery stenosis (36,37) (Figure 1).

## Plaque neovascularization

New vessel formation (neovascularization) is associated with plaque instability and vessel hypoxia (38,39). These vessels have low-velocity blood flow, which in conventional Doppler imaging is filtered out by a wall filter to remove clutter and motion artifacts, resulting in a loss of this low-flow component. Therefore, the limitation for conventional Doppler imaging to detect microvascularity makes the evaluation of intra-plaque neovascularization very challenging.

### Contrast enhanced ultrasound (CEUS)

CEUS has emerged as valuable ultrasound imaging modality that compliments and enhances standard ultrasound imaging mainly by providing an improved visualization of delineation of the vascular wall (the fibrous cap). Moreover, CEUS has been shown to reliably evaluate intraplaque neovascularization and identify carotid artery ulceration with superior diagnostic accuracy compared to the color Doppler techniques (40,41). Ultrasound contrast agents are sulforhexafluoride gas filled microbubbles measuring approximately 1-8 µm, which are injected intravenously and serve as intravascular tracers. These microbubbles contrast agents are stable, strong reflectors and resonators when exposed to an ultrasound beam. Contrast enhancement is usually graded on a 4-point scale. So far, quantification of contrast uptake within a plaque remains challenging, and methods for quantification are still under development. Several studies have demonstrated a good correlation of neovascularization detected by CEUS with microvessel density assessed by histological examination (40,42,43).

### Superb microvascular imaging (SMI)

SMI is a Doppler technology that allows the visualization of minute vessels with low flow signals without the use of contrast agents. SMI effectively separates flow signals from overlying tissue motion artifacts, preserving low-flow component (44).

## Plaque and artery stiffness

### Shear wave elastography (SWE)

SWE is an emerging ultrasound method, which exploits acoustic radiation forces to generate shear wave propagation in tissue. Recent research suggests that plaques rupture when the peak stress within the fibrous cap exceeds a certain threshold level (45). Knowledge about the stress distribution within the fibrous cap may therefore predict the risk of plaque rupture. A recent study by Garrard demonstrated that SWE was able to identify features of plaque vulnerability, and provide additional information related to plaque tissue characteristics (46). SWE enables the assessment of tissue stiffness by quantifying the Young’s modulus of elasticity.

### Transcranial Doppler

Transcranial Doppler monitors cerebral blood flow and can detect microembolic signals (MES) from the blood stream representing thromboemboli. Two large prospective studies showed that MES on transcranial Doppler (TCD) identified high-risk patients with ACS, and that patients with asymptomatic carotid stenosis and MES had a stroke risk comparable to symptomatic patients with high-grade stenosis (47).

### Computed tomography (CT)

The presence of clinically silent infarcts on CT brain imaging in patients with moderate to severe carotid stenosis (60%-79%) was in the ACSRS study found to be associated with an increased risk of stroke (48). CT brain imaging can therefore be of value in the risk assessment of asymptomatic carotid stenosis.

### Computed angiography (CTA)

CT angiography (CTA) is used in clinical practice for the assessment of plaque and degree of artery stenosis. Studies have found that the overall agreement on the estimation of the degree of artery stenosis between carotid Doppler ultrasound and CTA is good at 79.1% (95% confidence interval 0.72-0.83). However, CTA is unable to reliably distinguish between moderate (50%-69%) and severe (70%-99%) stenosis, which is important for clinical management and a limitation of this investigation. CT angiography, however, cannot accurately measure plaque volume (because remodeling can accommodate large plaques with little impact on lumen diameter) (49).

## Magnetic resonance imaging (MRI)

### Carotid MRI

Recent developments in MRI technology have shown promise allowing the identification high-risk carotid plaque characteristics (large LRNC, IPH, TRFC/ulceration) and the accurate discrimination between the specific histological subtypes of carotid plaques as proposed by the American Heart Association (50). Multiple single-center prospective trials have shown the ability of these multiple MR-defined plaque characteristics, such as the size of the LRNC, IPH, and TRFC to stratify risk of future carotid TIA or stroke (51,52). These high-risk plaque characteristics such as a large LRNC may represent a phenotype of asymptomatic carotid artery stenosis with high risk of future events that may be amenable to intensive medical therapy. IPH and ruptured FC/ulceration may represent vulnerable plaque that requires close monitoring to identify plaque progression or new symptoms despite intensive medical therapy and in some cases may require surgical intervention.

Gupta et al (51) in a systematic review that included 9 MRI studies with 779 participants found that carotid plaques with LRNC, IPH, and a thin, ruptured fibrous cap identified on MRI were significantly more likely to result in ipsilateral ischemic events. Four of the 7 studies examined more than one plaque element, but a multiparametric testing approach addressing the significance of each plaque component was not performed in any of the studies. The authors concluded that MRI characterization of specific plaque elements (LRNC, IPH, and TRFC) could provide additional measures of stroke risk not provided by simple measurements of luminal stenosis (51).

The majority of current imaging studies of atherosclerotic plaques rely on a human observer´s interpretation of the MRI findings with different contrast weighting, producing measurements that have been compared with histological assessments. This manual plaque segmentation requires expertise, is time consuming, and produces results that are subject to interobserver variability (53). Contrast enhanced automated classification may provide more objective and reliable assessments of plaque composition. Klooster et al (54) imaged 40 patients who had carotid plaques with MRI and found good agreement between automatic and visual identification of plaque components. They found that the volumes of hemorrhage and lipids assessed by visual and automatic assessments were reasonably consistent but not for calcium. In a further study semi-automated MRI assessments of the percentage of LRNC in carotid plaques were found to be significantly correlated with the percentage LRNC on histological assessment, and to echolucency on ultrasound, with echolucent plaques having larger LRCN compared to echogenic plaques (55).

MRI of plaque composition is a relatively new technique and studies have shown a high diversity in findings using different MRI protocols and different techniques in the histological assessments (51,56). It is therefore difficult to draw definite conclusions regarding the value of carotid plaque MRI characterization. In addition, patients are often included in the studies after a long time interval from symptom onset, and the possibility that the plaque components may change over time was therefore not taken into account.

### Brain MRI

Moderate and severe internal carotid stenosis has been associated with higher prevalence of clinically silent cerebral infarcts on brain MRI and this investigation may therefore aid risk assessment for patients with asymptomatic carotid stenosis (57).

## Molecular imaging

In recent years there has been growing interest in the ability of positron emission tomography (PET) to assess plaque inflammatory content. Due to the infiltration and retention of oxidized lipids in the arterial wall, vulnerable plaques contain a greater density of macrophages compared to asymptomatic plaques (58). Activated macrophages have a significantly increased metabolic rate and therefore increased 2-deoxy-2- [^18^F] fluoro-D-glucose (^18^F-FDG) uptake. Rudd et al (59) found increased ^18^F-FDG in macrophage-rich regions of carotid plaques, removed at endarterectomy, in 8 symptomatic patients compared to contralateral asymptomatic plaques in the same patients. Tawakol et al (60) demonstrated that in vivo ^18^F-FDG uptake correlated with the degree of carotid plaque inflammation in 17 patients when macrophage staining was assessed histologically.

^18^F-FDG uptake has also been shown to correlate with other factors that are associated with plaque instability. Carotid plaques with decreased ultrasound echogenicity and patients with increased serum lipids have been found to have higher degree of ^18^F-FDG uptake on PET (61-63). Evidence from longitudinal studies also suggests that arterial ^18^F-FDG uptake may be related to patient outcome. Figueroa et al (64) followed 513 patients without symptomatic cardiovascular disease for a mean of 4.2 years. They found that ^18^F-FDG uptake in the wall of the ascending aorta was an independent predictor of future cardiovascular events. Results from the Dublin Carotid Atherosclerosis Stroke Study showed, in 67 patients with a recent ischemic event (TIA or stroke), that carotid plaque inflammation, measured by ^18^F-FDG PET, was associated with a high risk of early stroke recurrence, independent of the degree of stenosis (65).

These studies have, however, been limited by relatively small sample sizes and time delays of weeks or months from symptoms to ^18^F-FDG PET imaging and histology following endarterectomy. There is therefore a possibility that plaque inflammation may have been modified by medications and life-style changes during these time-delays from symptoms to imaging and histological assessments. Increased ^18^F-FDG uptake must be closely correlated in time to ipsilateral ischemic cerebral events and be higher in symptomatic compared to asymptomatic patients if this method is to be of value in the clinical management of patients.

Combined MRI and PET can be expected to have an additional value over PET/CT in non-invasive imaging of atherosclerosis since CT does not visualize the vessel wall but primarily the lumen. Despite the limited spatial resolution of PET, CT can give the anatomical localization of the ^18^F-FDG signal with regard to individual atherosclerotic lesions. CT angiography, however, cannot accurately measure plaque volume because remodeling can accommodate large plaques with little impact on lumen diameter. A whole-body PET/MR imager allowing simultaneous MR and PET imaging has therefore been developed. The main advantage of this method is the perfect alignment between PET and MRI that allows for a precise delineation of the vessel wall, or plaque, and characterization of plaque components (eg, the necrotic core). In addition, it requires less examination time compared to sequential MRI and PET. In comparison with PET/CT the advantages of MRI are decreased radiation and superior visualization of soft-tissue plaque components.

Improvements in new-generation PET scanners are believed to provide better resolution and thereby better imaging of small structures such as carotid plaques. The use of macrophage specific tracers, 64-CU DOTATAE, 11-C-PK11195, are being evaluated with the hope of improving the plaque/background ratio of glucose (66). In addition to improving the assessment of inflammation in carotid plaques, these tracers may eventually allow the imaging of atherosclerosis in arteries where background uptake of ^18^ F-FDG would be prohibitively high, such as those in the cerebral and coronary circulation.

## Biomarkers of plaque instability

In addition to CRP many different inflammatory mediators involved in the atherosclerotic process have been measured in plasma and found to be associated with plaque instability (fibrinogen, leukocytes MMP-7, IL23, visfatin granzyme B CD-36 IL-6 VCAM-1). However, thus far no single biomarker has been found able to reliably predict ischemic stroke or CVD (67-74).

## Conclusion

Carotid plaque assessment with carotid ultrasonography and MR imaging provides superior risk stratification for individual patients compared with carotid stenosis and other epidemiologically identified cardiovascular risk factors. New methods in ultrasonography and molecular imaging can provide new information on plaque pathology and have the potential to aid in cerebrovascular risk assessment. Application of plaque imaging in prospective studies and multicenter trials in patients with asymptomatic and symptomatic carotid artery stenosis will hopefully, in the near future, tell us how we can best guide treatment on an individual patient level.
